# The Hospitals and Dispensaries of Bengal
**Triennial Report of the Working of Hospitals and Dispensaries under the Government of Bengal for the Years 1911, 1912, and 1913.* By Colonel G. F. A. Harris, C.S.I., M.D., V.H.S., I.M.S., Inspector-General of Civil Hospitals, Bengal


**Published:** 1915-05-08

**Authors:** 


### THE HOSPITALS AND DISPENSARIES OF BENGAL.*

THE Triennial Report of the Inspector-General of Civil Hospitals, Bengal, covers the years 1911, 1912, and 1913. The chief event recorded is, of course, the raising of the province to the status of a Presidency; but among the facts specially reported upon, those affecting the independent medical profession in the province are given prominence. General practitioners were affected by several schemes designed for their welfare, such as the reservation of the newly-created Chair of Anatomy in the Medical College for the members of that profession. In addition, a number of the resident and other medical posts in some of the larger Government State-aided and private hospitals can now be filled by non-service men. Opinions vary considerably as to the beneficial results of this arrangement, offering what one might think would be advantages likely to be keenly sought after and held by passed students of the Medical College and other practitioners. Lieutenant-Colonel Calvert, Principal of the Medical College, Calcutta, for instance, considers that the scheme has been accompanied by distinct loss of efficiency, control, and discipline in the hospital. Another alleged effect of the change is a constant tendency to increase expenditure.

The point of view of the service and non-service men on these matters is likely to be divergent, and the opinions quoted in the Triennial Report are generally those of service men. The writer of the Report, Colonel G. F. A. Harris, thinks that the scheme must be worked for a few years longer, but he considers that the analogy of large hospitals in Great Britain and elsewhere, which is sometimes adduced in favour of the system, is not applicable at present in India.

An additional measure adopted for the encouragement of the Indian medical profession was the association of selected members with the staffs of Government hospitals as honorary physicians and surgeons, allowing them facilities for consultation and the use of operating theatres. This innovation, however, is being cautiously proceeded with, and at present applies to only two of the Calcutta hospitals.

Another question closely affecting the interests of the Indian medical practitioners as well as the public was carefully considered in 1913; it was whether persons attending hospitals and dispensaries, maintained or assisted by public funds, should be considered to be entitled to gratuitous medical advice and treatment, irrespective of their circumstances. No fresh regulations, however, were issued in view of the inadvisability of doing anything that might hinder the growth of the popularity of the European system of medicine and medical treatment.

As in the Presidency there is a marked lack of efficient medical institutions other than the State-maintained and State-aided hospitals, and as association with these by the general practitioner is so grudgingly allowed, one would judge that the competition of the chief hospitals, where there is admittedly little or no inquiry as to the financial suitability of the patients, together with other conditions, make an adverse combination greatly to the disadvantage of the independent medical profession of Bengal.

The Report records the inauguration of a scheme for establishing a School of Tropical Medicine for the convenience of medical men, both official and non-official, who are anxious to study the subject but are unable to go to England for the purpose.

Many of the dispensaries mentioned in the Report, we gather, are in reality small hospitals with accommodation for both in- and out-patients. The number of hospitals and dispensaries in Calcutta classified in the Report is nineteen, seven of which treat out-patients only. The total number of intern patients at the twelve institutions with beds during the triennium was as follows:—28,983 in 1911, 31,480 in 1912, and 32,602 in 1913. The aggregate showed an increase of 9,134 over the preceding triennial period. It is thought that this indicates an increase of popularity with the public. Certainly much is done to adapt the institutions to local peculiar conditions; for instance, at the Calcutta Dufferin Hospital the staff of medical women has been strengthened, and some Brahmin pupil-nurses have been engaged. It is hoped that this step may induce more high-caste Hindu patients to seek the benefits of the hospital.

Improved nursing of patients has secured a large measure of attention. Prior to 1911 the nursing arrangements in the Medical College group of hospitals were in charge of the nursing branch of the Clewer Sisterhood. The service of these ladies is alluded to in high terms of appreciation, but, as they had not all received the thorough training in all branches of nursing now considered essential, the system was given up, and the work is now carried out by a qualified lady superintendent of nurses and a staff of nursing sisters "all of whom are fresh from London, the greatest centre of modern nursing training in the world."

Of 343,232 in- and out-patients treated at the Calcutta Hospitals, 49,882 were Europeans and Eurasians. At the Eden Hospital there has been a fall from 5,047 out-patients in 1911 to 3,008 in 1913, attributed to the presence of male students for the purpose of efficient teaching of gynæcology, which in India is especially a difficult and delicate matter.

Voluntary contributions to the institutions that are partly supported by the State formed only 1.13 per cent. of the total income, less than one-third of the amount received in the previous year. Very little is given by the Indian community towards maintenance, although large sums are from time to time conditionally offered by generous and wealthy natives for the purpose of building new hospitals.

* *Triennial Report of the Working of Hospitals and Dispensaries under the Government of Bengal for the Years 1911, 1912, and 1913.* By Colonel G. F. A. Harris, C.S.I., M.D., V.H.S., I.M.S., Inspector-General of Civil Hospitals, Bengal

